# In Vitro Fish Models for the Analysis of Ecotoxins and Temperature Increase in the Context of Global Warming

**DOI:** 10.3390/toxics9110286

**Published:** 2021-11-01

**Authors:** Bianka Grunow, George Philipp Franz, Katrin Tönißen

**Affiliations:** Research Institute for Farm Animal Biology (FBN), Institute of Muscle Biology and Growth, 18196 Dummerstorf, Germany; franz@fbn-dummerstorf.de (G.P.F.); toenissen@fbn-dummerstorf.de (K.T.)

**Keywords:** fish cells, 3R, sturgeon, salmonid, ecotoxicology, chemical solvents, climate change, ocean warming

## Abstract

Rising temperatures can affect fish survival, especially from shallower waters, as temperatures increase faster and more intensively in these areas; thus, species-specific temperature tolerance can be exceeded. Additionally, the amounts of anthropogenic pollutants are higher in coastal waters. Although increasing metabolic activity at higher temperatures could lead to stronger effects of toxins, there are hardly any studies on this topic. Subsequently, the aim was to investigate the response of fish cells upon exposure to industrial solvents (ethanol, isopropanol, dimethyl sulfoxide (DMSO)) in relation to a temperature increase (20 °C and 25 °C). Concerning the 3Rs (the replacement, reduction and refinement of animal experiments), in vitro tests were used for two threatened, vulnerable fish species: maraena whitefish (*Coregonus maraena*) and Atlantic sturgeon (*Acipenser oxyrinchus*). Both cell lines exhibited higher proliferation at 25 °C. However, ecotoxicological results indicated significant differences regarding the cell line, toxin, temperature and exposure time. The evolutionarily older fish lineage, Atlantic sturgeon, demonstrated lower mortality rates in the presence of isopropanol and recovered better during long-term ethanol exposure than the maraena whitefish. Atlantic sturgeon cells have higher adaptation potential for these alcohols. In summary, fish species respond very specifically to toxins and changes in temperature, and new ecotoxicological questions arise with increasing water temperatures.

## 1. Introduction

It is well-documented that climate change widely affects aquatic habitats. Particularly in shallow water bodies, which possess higher biodiversity and provide spawning grounds for many fish species, climate change leads to higher temperatures. Thus, development and survival are challenging for mostly ectotherm fish because they are forced to survive in increasingly extreme environments [1]. Furthermore, oxygen solubility decreases with higher water temperature, resulting in hypoxia in aquatic systems [2,3,4]; additionally, toxins, anthropogenic pollutants, and the resulting eutrophication decrease the survival chances for many species [5,6,7].

It is predicted that, in 2100, the average global surface temperature will increase by about 5 °C, as expected by climate simulation models [8,9]. However, some aquatic organisms from shallow waters have already reached their thermal tolerance limits, which creates the potential for the negative effects on their development, growth, metabolism and survival rate even more [4,10,11,12,13]. Mobile organisms such as pelagic fish are still able to move into different areas to evade higher temperatures and unsuitable water parameters. However, site-faithful fish, spawners, and fish in less-mobile developmental stages are forced to deal with these harsh conditions [14,15].

The effects of higher temperatures on species are a main research focus. However, how do aquatic organisms react to chemical pollutants when water temperatures increase? Additionally, does temperature affect bioavailability or bioaccumulation? Is there a higher environmental risk for aquatic organisms due to increasing temperatures?

To address these questions, fish cells were used as an animal-free alternative to the frequently applied ecotoxicological method of in vivo lethality tests with live fish [16]. Following classical toxicological testing approaches, the assessment focused on endpoints (mortality) at both the organism and cellular levels [17,18,19]. The measurement of mortality is ecologically relevant because it can influence the overall growth rates of populations. The application of treatments on cells presents a valid comparison because the chemical concentration that causes cell death in vitro would also cause cell death in vivo, as it would consequently result in a lethal systemic failure of the organism [20].

On the basis of this background, the effects of three chemicals were observed in more detail. According to information from the European Chemicals Agency (ECHA) and the U.S. Environmental Protection Agency Committee (EPA), chemicals ethanol and isopropanol are classified as non-toxic, and dimethyl sulfoxide (DMSO) is classified as of low concern [21]. Both agencies are a driving force among regulators in implementing chemical legislation in the European Union and United States to protect human health and the environment, and to promote innovation and competitiveness.

All three chemicals are industrially used as solvents. Alcohols such as ethanol and isopropanol are in especially high demand [22], and are some of the oldest organic processes utilized by humankind. Although ethanol and isopropanol are not classified as hazardous, they can cause major environmental damage because of their good solubility in water. This environmental threat is exacerbated by growing bioethanol production [23]. Examples of large-volume ethanol spills and environmental impact can be found on several continents, as shown by examples in the Peene River, northeast Germany, in 2015 [24] and in Kentucky, USA [23].

DMSO, as a polar solvent, exhibits high solubility for many polymers, and may aid in polymer processing and clean-up. However, in small concentrations, DMSO is also naturally formed and is mainly derived from the photochemical and bacterial oxidation of dimethyl sulfide (DMS), and from direct synthesis in marine algal cells [25,26]. Measurements of DMSO from 2009 to 2018 in the southwestern Baltic Sea revealed that significant interannual and seasonal variations in mixed-layer concentrations are present, with values between 2.5 and 209.8 nmol L^−1^ [27].

Due to new chemical regulations such as the European Registration, Evaluation, Authorisation and Restriction of Chemicals (REACH) and a constantly growing number of chemicals in everyday use, the need to test for their bioactivity keeps growing, and in vitro models are now indispensable. Tannenberger et al. (2013) demonstrated the possibility to switch from in vivo to acute in vitro toxicity testing because there was almost 1:1 correlation between the conventional embryo test and the use of fish cells in the evaluation of 35 components [28]. In order to receive an Organisation for Economic Co-operation and Development (OECD) guideline for fish cell lines and thus be legitimately used in ecotoxicology, an International Organization for Standardization (ISO) guideline (ISO21115:2019) was adapted for rainbow trout gill cell line RTgill-W1 in 2019 [20,29]. Due to the large range of species (~33,000 [30]) and their diverse evolutionary lineages, fish also differ in their ecological background, and thereby in their physiology. Consequently, temperature tolerances vary between species. Therefore, fish also respond to environmental changes and chemical inputs, depending on various evolutionary adaptations. The goals of this study were to investigate the effects of elevated temperature on toxicity in an in vitro model. For this, two fish cell lines established from vulnerable, threatened species were chosen, because both species are particularly affected by temperature increases. The first cell line was obtained from the maraena whitefish, and the second from the Atlantic sturgeon.

The maraena whitefish (*Coregonus maraena*, Bloch, 1779), like the well-studied rainbow trout (*Oncorhynchus mykiss*, Walbaum, 1792), belongs to the salmonids and is an ecologically important species in the Baltic Sea region [31]. Maraena whitefish, which spawns mainly in estuaries, were brought to the edge of extinction in the 20th century. The causes were extensive fishing, and also habitat fragmentation due to construction processes and eutrophication due to high agricultural inputs. However, intensive restocking operations in the 1990s led to a short-term stabilization of the population [32,33]. Studies in aquaculture showed that maraena whitefish optimally grow at water temperatures between 12 and 18 °C, while temperatures above 22 °C impair growth, and lethal temperatures are reached above 26 °C [34,35]. The whitefish, in the case of an acute temperature change from 18 to 24 °C, reacts less stress-induced than the animals that experienced a temperature warming over a longer time period [35]. Therefore, whitefish may be heavily affected during the gradual temperature increase that occurs due to climate warming. Ficker et al. (2016) calculated that a period of 50 years increasing water temperatures resulting from global warming would reduce the whitefish biomass of wild populations by between 3% and 8% [36].

The Atlantic sturgeon (*Acipenser oxyrinchus*, Mitchill, 1815), a member of the Acipenseridae, is globally threatened and is extinct in Europe due to massive overfishing, damming, river regulation, and pollution [37]. Due to the very long life cycles, restocking operations are tedious [38]. Climate change could destroy this initial success, as temperature and dissolved oxygen are the main influencing factors for spawning and larval development in *A. oxyrinchus* [39,40,41,42]. The temperature increase shows a stronger influence on the spawning and development of the fish. Adult fish might be able to avoid extreme temperatures by a habitat change [43]; fish eggs or their larvae are in many cases dependent on shallow water areas. These habitats have a stronger temperature increase and resulting lower dissolved oxygen levels. Both are key habitat parameters for Atlantic sturgeon development [40,41,42,44,45,46], as larvae hatch from their adhesive eggs about 4–6 days after deposition at temperatures between 20 and 18 °C [45,47,48]. Another aspect of increased water temperatures affects food availability. For example, plankton growth strongly depends on the light cycle, temperature-induced premature spawn would result in a lack of food sources for larvae. Conclusively, the population tends to decrease.

This study investigates how the cells of these two evolutionary different non-model fish species react to a 5 °C increase projected in the climate models [8,9]. Additionally, we analyse how the reaction to the exposure to ecotoxicological relevant industrially used chemicals changes under different temperature regimes.

## 2. Materials and Methods

### 2.1. Cell Culture

A cell line from Atlantic sturgeon larvae (*A. oxyrinchus*; AOXlar7y [49]) was obtained from the German Cell Bank for Wildlife (Fraunhofer EMB, Lübeck, Germany) and cultivated for several passages before use. The cell line from maraena whitefish (*C. maraena*) was generated from fin tissue (CMAfin1) following the protocol from Grunow et al. (2011) [49]. Within these experiments, passages of P18–20 (CMAfin1) and P26–P28 (AOXlar7y) were used. Both cell lines exhibit markers for stem cells, mesenchymal cells, fibroblasts, and markers of the cytoskeleton. Cell lines were cultivated in Leibovitz-15 Medium, (L-15, Gibco, BioScience; Dublin, Ireland) with 10% fetal bovine serum (FBS) and 1% (*v*/*v*) penicillin/streptomycin at 20 °C. When cell confluence of 90% was reached, cells were passaged with a standard trypsination protocol. For this, cells were washed with 1× Dulbecco’s phosphate buffered saline (DPBS), followed by 2 min trypsination with 0.1% trypsin/EDTA solution at room temperature for CMAfin1 or at 37 °C for AOXlar7y. Trypsination was stopped by adding at least a double amount of the cell culture medium. Cells were centrifuged for 5 min at 130 rcf (Lisa Centrifuge 2 L, AFI MultiLab) and subcultivated at a ratio of 1:2 to 1:3. Cell number was measured with the use of the Eve™ Plus EU Automated Cell Counter (NanoEntek Inc., Seoul, Korea) following the manufacturer’s instructions.

### 2.2. Ecotoxicological Studies Regarding Climate Change

For this approach, the Incucyte^®^ S3 Live-Cell Analysis System (Sartorius AG; Göttingen, Germany) was used, as live-cell imaging permits cell proliferation to be monitored in real time over a period of several days. This system was successfully established for several applications, including toxicological studies [50,51,52]. Initial, 0.75 × 10^4^ cells of each cell line were added per well of a 96-well plate. Cultivation was performed at 20 and 25 °C. After cell attachment (6 h), chemical solvents with a wide range of industrial uses were added at different concentrations prepared in culture medium. The chemicals were: I. CH3CH2OH (ethanol): 4.0%, 2.0%, 1.5%, 1.0%, 0.5%, 0.25%; II. (CH3)2CHOH (2-propanol-isopropanol): 2.0%, 1.0%, 0.5%, 0.25%, 0.125% and III. (CH3)2SO (dimethyl sulfoxide; DMSO): 20%, 10%, 5%, 2.5% 1.0%, 0.5%. Cells within the control were cultivated with standard cell culture conditions without chemical addition. The control group was used to examine growth behaviour at different temperatures, and to calculate the differences in growth between the control and chemical groups. All experiments were performed in four replicates. Cells were observed over 65 h. Two time periods were evaluated. Acute toxicity was calculated as the mean of the measured values (% mortality) of the first 3.5 h directly after chemical addition. During this time, an intensive response was found in the cell growth curves. Long-term toxicity in this study is defined as mean values (% mortality) of the last 6 to 8 measurements (time between hours 48 and 65) of the experiment.

### 2.3. Image Analysis and Statistic

**Figure 1:**
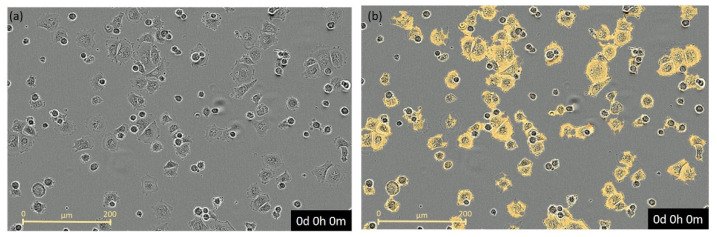
Cells of Atlantic sturgeon (**a**) in phase contrast and (**b**) with overlain masked (yellow) of attached cells for analysis of confluence generated by Incucyte^®^ real-time video imaging system (Sartorius AG).

Three to four images per well were taken every 30 min during the first 24 h of the experiment, and afterwards every 2 h in the Incucyte^®^ Zoom HD/2CLR time-lapse microscopy system (Essen BioScience, 2018) equipped with an Incucyte^®^ Zoom 20× Plan Fluor objective (Sartorius AG) in phase contrast (Figure 1a). Time-lapse videos were generated for every experimental condition. Representative images were used to train the Incucyte^®^ real-time video imaging system (Sartorius AG) to identify cells within different morphologies for later analysis regarding proliferation or confluence (yellow marked cells, Figure 1b). To find the confluence of each experimental trial, the mean and standard error of the mean (SEM) of each well was first calculated from the three to four images taken per well. Second, a single mean growth curve for each experimental trial was calculated on the basis of the four replicates for later illustration and final analysis. In the toxicological approaches, data were normalized to the time point of chemical addition. Similarly, percentage change in confluence was set to the confluence value before the addition of the chemical for the calculation of mortality. All data were expressed as the mean ± SEM. For all in vitro assays, Gaussian distribution was checked using the Shapiro–Wilk test, and acute and long-term toxicity was analysed with the Kruskal–Wallis test followed by a Dunn’s test for multiple comparisons. Significant differences were defined by *p* < 0.05 calculated in Graphpad Prism Version 9.0.2 and SAS software version 9.4 (Statistical Analysis Institute Inc., Cary, NC, USA).

## 3. Results

### 3.1. Faster Cell Proliferation at Higher Temperatures

**Figure 2:**
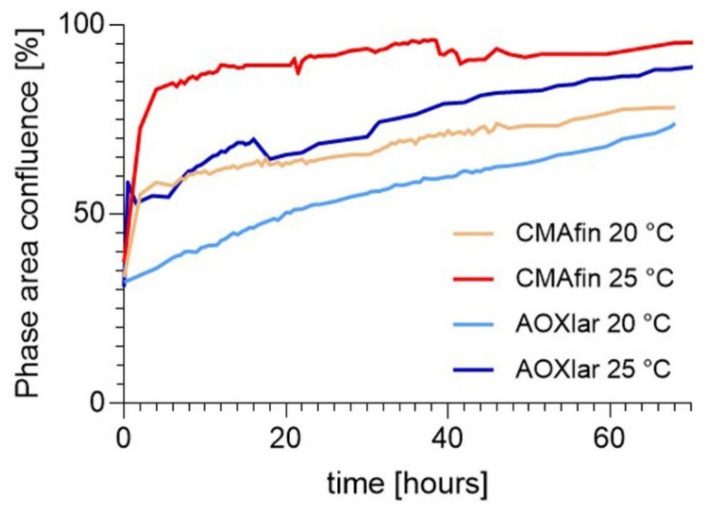
Proliferation of cells cultivated with different temperatures (20 and 25 °C). Growth rates were measured using an Incucyte^®^ real-time video imaging system (Sartorius AG). Blueish curves are cells of the Atlantic sturgeon (AOXlar7y), and reddish lines are cells from maraena whitefish (CMAfin1).

The experiment at different temperatures showed that cells reached confluence significantly faster at 25 °C (Figure 2). In more detail, cells cultivated at 25 °C attached two hours after seeding, whereas cells at 20 °C reached attachment 6 h after seeding. In addition, it was evident how quickly the cells of maraena whitefish (CMAfin1) grew in the higher-temperature setting (Figure 2). At the end of the experimental time, CMAfin1 cells cultivated at 25 °C had a confluence of 92.27 ± 3.5% and AOXlar7y of 85.90 ± 2.8%. Compared to the higher temperature, cells cultivated at 20°C exhibited nearly 20% less confluence within the wells after these 2.5 days of cultivation (CMAfin1: 76.65 ± 2.8% and AOXlar7y: 67.84 ± 4.3%; Figure 2).

### 3.2. Response to Ethanol

**Figure 3:**
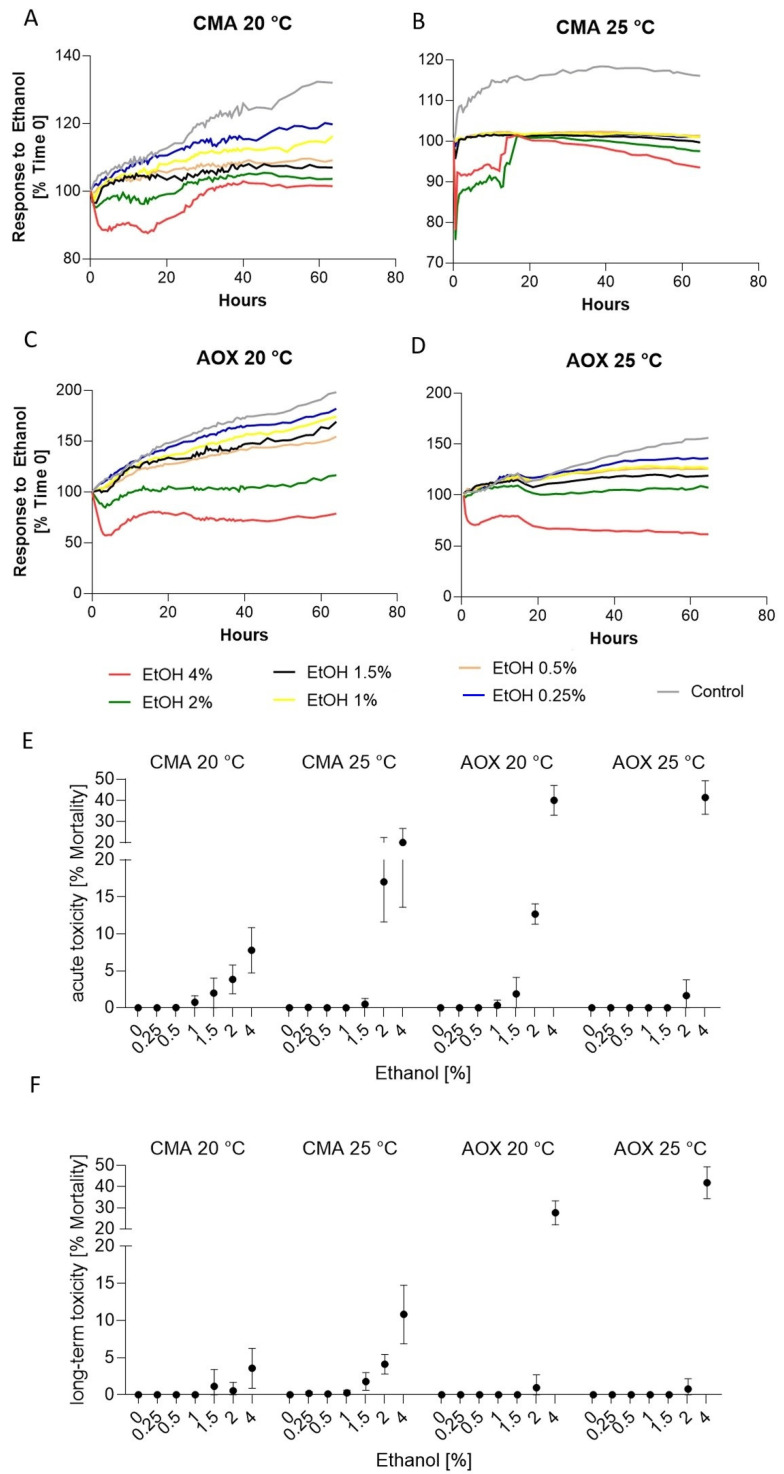
Response of CMAfin1 (*C. maraena*) and AOXlar7y (*A. oxyrinchus*) cells on ethanol addition under 20 and 25 °C cultivation. (**A**–**D**) Visualization of growth curves over time (% confluence) at different ethanol concentrations (4%—red, 2%—green, 1.5%—black, 1%—yellow, 0.5%—beige, 0.25%—blue, control; 0%—grey). (**E**) Calculated acute toxicity (% mortality compared to the initial confluence value) and (**F**) long-term toxicity (% mortality compared to the initial confluence value) from 4 replicates per experimental trial.

Analyses indicated that there were differences in the course of the curves, and thus the response to the ethanol input of the cell lines and regarding the two temperatures (Figure 3 and Figure S1). In all four experimental approaches, the cells reacted most strongly to the two highest concentrations of 4% and 2% ethanol. At a 4% ethanol level, CMAfin1 cells cultured at 20 °C showed an initial drop of slightly over 10% during the first 18 h after addition, but no cell death was visible (Figure 3A,F). Afterwards, CMAfin1 cells recovered or at least became accustomed, and proliferation resumed. However, cells were very granular at the end of the observation period (Figure S1b). In the 2% ethanol concentration, on the other hand, the drop was only just under 5%, but significantly different to the control (Figure 3F, Table 1). Recovery also began within the first 24 h; at the end of the study period, there were no significant differences related to the control (Table 1).

The reaction during 25 °C cultivation was significantly more intense compared to the 20 °C treatments (Table 1, Figure 3B,E,F). After 4% and 2% ethanol addition, an immediate 20% decrease in cell confluence occurred (Figure 3F). Additionally, cell structures became very rounded (Figure S1c). Recovery of acute stress occurred around 20 h, but compared to the control and lower ethanol concentration, more CMAfin1 cells died during the course of the trials (Figure 3B and Figure S1d).

Analysis of the AOXlar7y cell reaction indicated only a significant response at 4% ethanol addition (Figure 3C–F, Table 1). At 20 °C, the AOXlar7y cells dropped down to 57.57 ± 2.7% confluence (Figure 3C) and exhibited 40.06 ± 3.5% mortality at acute toxicity (Figure 3F and Figure S1e). AOXlar7y cells appeared to slightly recover over the course of the time (Figure S3g) and showed long-term mortality of only 27.68 ± 2.8% (Figure S3g). Cells even started to proliferate at the end of the observation and did not develop a granular structure as found in the CMAfin1 cells (Figure S1f). At 25 °C, cells reacted significantly more strongly compared to 20 °C, so that the confluence decreased with time. In the end, long-term mortality of 41.88 ± 3.3% was determined (Figures S1g,h and S3d,g). Lower concentrations did not significantly affect cells in their confluence and thus in their growth behaviour.

In total, AOXlar7y cells responded significantly more strongly and both cell lines reacted more intensely to ethanol with a higher cultivation temperature (Table 1).

### 3.3. Response to Isopropanol

**Figure 4:**
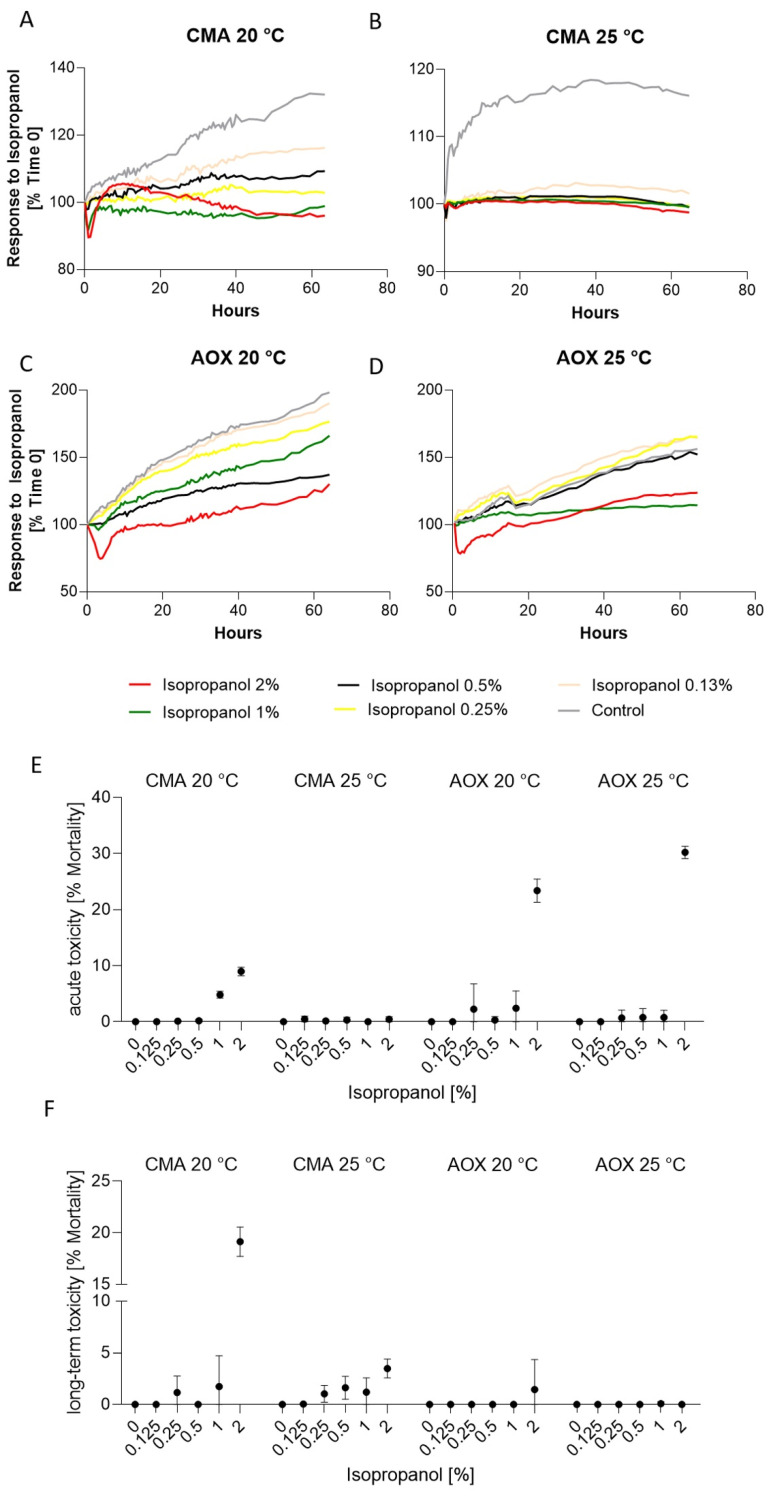
Response of CMAfin1 (*C. maraena*) and AOXlar7y (*A. oxyrinchus*) cells on isopropanol addition under 20 and 25 °C cultivation. (**A**–**D**) Visualization of growth curves over time (% confluence) at different isopropanol concentrations (2%—red, 1%—green, 0.5%—black, 0.25%—yellow, 0.125%—beige, control; 0%—grey). (**E**) Calculated acute toxicity (% mortality compared to the initial confluence value) and (**F**) long-term toxicity (% mortality compared to the initial confluence value) from 4 replicates per experimental trial.

Examining the response to increasing isopropanol concentrations (Figure 4) showed that, in acute toxicity, AOXlar7y cells especially showed a significantly stronger response at both temperatures (Figure 4E), whereas in the assessment of the final state, mainly CMA1fin cells (Figure 4F) had elevated mortality. Thus, at 25 °C and a concentration of 0.25% isopropanol, significant differences could be determined in comparison to the AOXlar7y cells. However, the highest investigated concentration of 2% isopropanol was significantly more severe for CMAfin1 cells at 20 °C, with a long-term toxicity of 19.14 ± 1.0% (Figure 4F, Table 2).

CMAfin1 cells at 20 °C generally reacted in different ways compared to the three other experimental groups. CMAfin1 cells with the lowest indication of 0.13% isopropanol proliferated and showed a confluence increase to 116.2 ± 5.8%. Cells with a 0.25% isopropanol addition stagnated and showed an average confluence increase of only 2.85%. Compared to this stagnation, cells cultivated with 0.5% isopropanol addition revealed higher proliferation of 9.29 ± 3.4%. With a 1% indication, cell confluence decreased to 91.96 ± 0.3% after one hour, and stagnated slightly below their initial value until the end of the trial. A 2% isopropanol indication caused a fast decrease during the first hour, which seemed to recover afterwards for the next few hours, but long-term toxicity was obvious (Figure 4A,F). The growth of AOXlar7y cells was present at both 20 and 25 °C (Figure 4C,D and Figure S2e–h), although at 25 °C, growth was lower or even stagnated at the two highest concentrations (confluence at 2% isopropanol: 123.94 ± 9.5% and 1% isopropanol: 114.38 ± 5.9%). In the two lowest isopropanol concentrations, cell proliferation was similar or even slightly higher compared to the control group (Figure 4C,D).

### 3.4. Response to Dimethyl Sulfoxide (DMSO)

**Figure 5:**
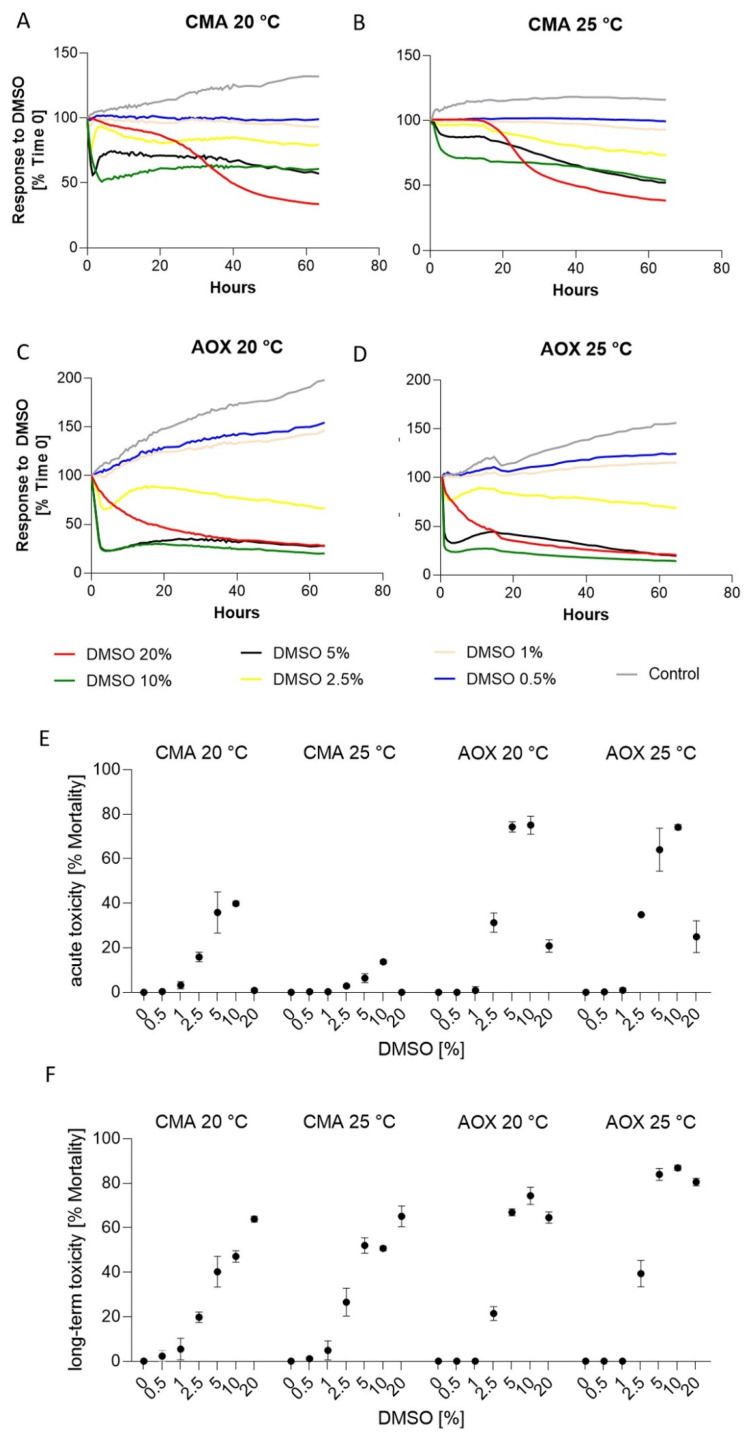
Response of CMAfin1 (*C. maraena*) and AOXlar7y (*A. oxyrinchus*) cells on DMSO addition under 20 and 25 °C cultivation. (**A**–**D**) Visualization of growth curves over time (% confluence) at different DMSO concentrations (20%—red, 10%—green, 5%—black, 2.5%—yellow, 1%—beige, 0.5%—blue, control; 0%—grey). (**E**) Calculated acute toxicity (% mortality compared to the initial confluence value) and (**F**) long-term toxicity (% mortality compared to the initial confluence value) from 4 replicates per experimental trial.

The addition of DMSO indicated how differently the two cell lines could react to the same treatment (Figure 5, Table 3). Analysis of the acute toxicity indicate a more intense response of the CMAfin1 cells at 20 than at 25 °C (Figure 5A,B) where significant differences were already present at 2.5% DMSO compared to the control (Table 3).

Further observation of the CMAfin1 cells indicated long-term decrease at 20% DMSO (Figure 5A,B). CMAfin1 cells were in a very fragile state with confluences of only 33.72 ± 0.5% (20 °C) and 39.25 ± 2.5% (25 °C) compared to the initial value. Significant differences between the two CMAfin1 temperature groups occurred at 5% and 10% DMSO (Table 3). Additionally, CMAfin1 cells seemed to recover more quickly at 20 than at 25 °C (Figure S3a–h).

Analysis of AOXlar7y cells showed no significant differences between two temperature groups in acute toxicity, but in long-term toxicity beginning at 2.5% DMSO upwards (Table 3). At the two lowest DMSO concentrations (0.5% and 1.0%), AOXlar7y cell proliferation was unaffected by acute and long-term toxicity (Table 3), with an increasing cell confluence at 20 °C (1% DMSO: 149.93 ± 4.2%, 0.5% DMSO: 156.95 ± 15.7%) and at 25 °C (1% DMSO: 115.46 ± 8.3%, 0.5% DMSO: 124.57 ± 12.1%); (Figure S3l,p). AOXlar7y cells had a more pronounced reaction at 25 °C and exhibited less cell survival. At the end of the observation, only cell debris or detached cells were present (Figure S3i,j,m,n).

In total, in all four experimental groups, confluences decreased at the three highest DMSO concentrations (20%, 10%, 5%), but unlike AOXlar7y cells, CMAfin1 cells had significantly lower mortality values (Figure 5, Table 3).

## 4. Discussion

### 4.1. Effects of Temperature Increase

The examination of two different cell lines kept at different temperatures showed that cells reached confluence significantly faster at 25 than at 20 °C. It has been shown, that fish grow faster with increasing temperature, due to the Van ’t Hoff rule, but also that fish are negatively affected by rising temperatures in terms of reproduction [15,53], behaviour [54], and cellular physiology [55,56]. The occurring thermodynamic effects and chemical reaction kinetics that follow higher temperatures can trigger stress responses in the animals [57,58,59]. Adequate stress responses are initiated by the activation of physiological endocrine systems, predominantly about the release of the neurotransmitter cortisol and catecholamines to cope with these environmental changes (reviewed by [60]). This increases the level of complexity of temperature-related reactions, as illustrated in the review by Alfonso et al. (2020) [55]. Despite the activation of stress genes and a consequent negative effect on the immune system, higher temperatures can positively lead to faster fish growth [61,62]. This faster growth effect, as observed in our two cell lines with a significant higher proliferation at 25 °C, is already used in aquaculture to produce higher yields [63]. Whether cells were exposed to physiological stress despite the increased proliferation rate will be investigated in further studies.

### 4.2. Effect of Ethanol and Isopropanol

The toxicity tests of the ECHA indicate that generalized statements should be avoided even within one chemical class (alcohols) as the animals reacted with different intensities in the ethanol and isopropanol toxicity tests. In previous examinations of ethanol effects for the in vivo fathead minnow (*Pimephales promelas*, Rafinesque, 1844) a lethal concentration which causes the death of 50% (LC50) was identified for 15.3 g/L [64] whereas for rainbow trout an LC50 of 13 g/L was determined (ECHA, Dossier 16105/6/2/2, [65]). From various toxicity studies of isopropanol, we know that the LC50 is approximately 9 to 10 g/L (ECHA, Dossier 15339/6/2/2, [66]). The results obtained here indicate a difference in the responses. Like the in vivo data found in literature, the cells examined here tended to be also more sensitive to isopropanol than to ethanol. A comparison of both cell lines revealed a higher sensibility of the Atlantic sturgeon cells (AOXlar7y) in the short-term exposure of isopropanol, whereas the maraena whitefish cells (CMAfin1), reacted more intense in the long-term exposure. Altogether, several factors as species or possible differences between in vivo and in vitro models could influence the mortality. Similarly, Vera et al. (2018) described how ethanol toxicity as well depends on the time of day [67]. In zebrafish larvae, mortality rates of 82% in the morning and just 6% at night were measured after one hour exposure to 5% ethanol. Nevertheless, in the cells of both fish species a significant growth in mortality with increasing temperatures was observed. The analysis of the isopropanol toxicity differs in the cell response in the ethanol experiments. In the acute toxicity, the Atlantic sturgeon cells reacted most intensively comparable to the ethanol trials. Examining the long-term toxicity effects, the cells of the maraena whitefish were the most affected, and here the cells that were cultured at the lower temperature (20°C). The reason why the cells died more often at lower temperatures (also slightly the case with Atlantic sturgeon) remains unknown. References to this phenomenon could not be found in the literature.

### 4.3. Effects of DMSO

Organic sulfoxide DMSO is commonly utilised as a solvent in industry but also for cryopreservation. According to the ECHA, DMSO is of low concern for all kind of aquatic organisms (fish, invertebrates, algae). For fish, LC50 is species-specific and was determined at 25 to 43 g/L on the basis of a wider taxonomic sampling comprising zebrafish, fathead minnow, rainbow trout, green sunfish (*Lepomis cyanellus*, Rafinesque, 1819), bluegill (*Lepomis macrochirus* Rafinesque, 1810), and channel catfish (*Ictalurus punctatus*, Rafinesque, 1818) (ECHA, Dossier 15007/6/2/1, [68]). These toxicities are comparable to the results of CMAfin1-cells under the control condition at 20 °C. However, as an increase of 5 °C led to significantly higher mortalities, cells were already exposed to physiological stress due to the higher temperature and, therefore, reacted more sensitively to the chemical. The Atlantic sturgeon reacted even more intensely to a DMSO addition. In principle, sturgeon cell mortalities are significantly higher under control conditions (without chemical addition) at a higher temperature compared to the examined salmonid species at 25 °C. With an increase in temperature and simultaneous exposure of 5–20% DMSO, nearly 100% mortality was present in the sturgeon cells. Several studies examined the effect of DMSO on chondrocytes (in mammals), showed that higher temperatures increased the toxic effect, and labelled 1M DMSO as starting to generally be toxic to cartilage cells [69] and for other cell types already at 0.5% [70]. As sturgeon skeletons remain cartilaginous over the course of their lives, these studies could be especially relevant to this extraordinary fish family.

The previous literature on temperature-related effects of chemical toxicity remains sparse, but they were acknowledged in some early ecotoxicological studies. Especially for DMSO, temperature had a profound effect on survival [71]. In the examined salmonids, besides death, abnormal haematocrit levels and the abnormal cell morphology of several organs (liver, kidneys, gills, spleen, brain, blood) occurred at high DMSO concentrations and/or after prolonged exposure [71,72]. While these studies allow for a comparison with the results obtained, the typical exposure period of fish cells in ecotoxicological screenings is typically limited to 24 h, and chemical toxicity is mainly evaluated as basal cytotoxicity resulting in cell death [73,74]. This approach delivers an improved comparison to effects in habitats, since pollutant entry, for example, due to spills, is connected with longer exposure times on lower-level concentrations. What influence the naturally formed DMSO in the water has on the fish has not yet been investigated. Due to the low concentration in nmol ranges, the influence of the occurring DMSO on fish physiology could be insignificant.

## 5. Conclusions

This current study demonstrates that higher water temperatures could cause higher cell proliferation. Concerning toxicity, no general statement on the ecotoxicological reactions in relation to temperature can be given. This study exhibits species-specific differences in relation to the solvent, exposure time, and temperature. Therefore, it is worth considering a re-evaluation of lethality studies. Even with the three chemicals classified as non-hazardous or of low concern that were used in this study, profound toxic effects occurred as a function of species and temperature. Additionally, the whitefish and the sturgeon react differently in respect to temperature and chemical substance. Previous studies showed that no generalised statements on “fish” can be provided, and that differences between orders, families, and genera exist (please see ECHA dossiers); smaller changes might also appear in interpopulation level comparisons. Here, only two species from two different orders of around 68 orders in the ray-finned fish (*Actinopterygii*) [30] were observed, and differences were obvious. As fish biodiversity is still commonly underestimated even though it represents approximately half of all vertebrates, larger taxon sampling for toxicological studies is necessary and cannot solely rely on a few model organisms. In this context, the usage of different species’ cell lines is even more important to reduce the increasing number of animals. In summary, ecotoxicological research needs to be expanded in light of climatic changes, and many questions remain open with respect to water ecosystems.

## Figures and Tables

**Table 1:** Statistical evaluation of percentage mortality rates due to increasing ethanol concentrations in intra- and interspecies comparison of Atlantic sturgeon (AOXlar7y, AOX) and maraena whitefish (CMAfin1, CMA) at cultivation temperatures of 20 and 25 °C. Significant different *p* values (<0.05) highlighted in red.

Ethanol [%]		CMA 20 °C (Compared to the Control)	CMA 25 °C (Compared to the Control)	CMA 20 °C vs. CMA 25 °C	AOX 20 °C (Compared to the Control)	AOX 25 °C (Compared to the Control)	AOX 20 °C vs. AOX 25 °C	CMA vs. AOX at 20 °C	CMA vs. AOX at 25 °C
acute toxicity	0.25	>0.999	>0.999	0.251	>0.999	>0.999	>0.999	>0.999	0.251
	0.5	>0.999	>0.999	0.419	>0.999	>0.999	>0.999	0.252	0.356
	1.0	0.936	>0.999	0.141	>0.999	>0.999	0.356	0.511	>0.999
	1.5	0.16	0.514	0.217	>0.999	>0.999	0.135	0.951	0.256
	2.0	0.036	0.022	0.006	0.051	0.464	<0.0001	<0.0001	0.003
	4.0	0.002	0.026	0.028	0.004	0.005	0.845	<0.0001	0.100
long-term toxicity	0.25	>0.999	>0.999	0.172	>0.999	>0.999	>0.999	>0.999	0.172
	0.5	>0.999	>0.999	0.153	>0.999	>0.999	>0.999	>0.999	0.153
	1.0	>0.999	>0.999	0.192	>0.999	>0.999	>0.999	>0.999	0.192
	1.5	>0.999	0.11	0.623	>0.999	>0.999	>0.999	0.356	0.024
	2.0	>0.999	0.015	0.006	0.904	>0.999	0.890	0.707	0.022
	4.0	0.028	0.001	0.023	0.002	0.001	0.023	<0.0001	<0.0001

**Table 2:** Statistical evaluation of percentage mortality rates due to increasing isopropanol concentrations in intra- and interspecies comparison of Atlantic sturgeon (AOXlar7y, AOX) and maraena whitefish (CMAfin1, CMA) at cultivation temperatures 20 and 25 °C. Significant different *p* values (<0.05) highlighted in red.

Isopropanol [%]		CMA 20 °C (Compared to the Control)	CMA 25 °C (Compared to the Control)	CMA 20 °C vs. CMA 25 °C	AOX 20 °C (Compared to the Control)	AOX 25 °C (Compared to the Control)	AOX 20 °C vs. AOX 25 °C	CMA vs. AOX at 20 °C	CMA vs. AOX at 25 °C
acute toxicity	0.125	>0.999	0.207	0.099	>0.999	>0.999	>0.999	>0.999	0.099
	0.25	>0.999	>0.999	0.725	>0.999	>0.999	0.534	0.377	0.469
	0.5	0.904	0.639	0.672	>0.999	>0.999	0.600	0.728	0.593
	1.0	0.018	>0.999	<0.0001	>0.999	>0.999	0.364	0.176	0.271
	2.0	0.016	0.146	0.001	0.007	0.016	0.025	0.003	<0.0001
long-term toxicity	0.125	>0.999	>0.999	0.198	>0.999	>0.999	>0.999	>0.999	0.198
	0.25	0.874	0.361	0.888	>0.999	>0.999	>0.999	0.195	0.044
	0.5	>0.999	0.065	0.026	>0.999	>0.999	>0.999	0.356	0.026
	1.0	>0.999	0.172	0.326	>0.999	0.416	0.356	0.203	0.165
	2.0	0.018	0.004	0.001	0.416	>0.999	0.356	0.001	0.001

**Table 3:** Statistical evaluation of percentage mortality rates due to increasing dimethyl sulfoxide (DMSO) concentrations in intra- and interspecies comparison of Atlantic sturgeon (AOXlar7y, AOX) and maraena whitefish (CMAfin1, CMA) at cultivation temperatures 20 and 25 °C. Significant different *p* values (<0.05) highlighted in red.

DMSO [%]		CMA 20 °C (Compared to the Control)	CMA 25 °C (Compared to the Control)	CMA 20 °C vs. CMA 25 °C	AOX 20 °C (Compared to the Control)	AOX 25 °C (Compared to the Control)	AOX 20 °C vs. AOX 25 °C	CMA vs. AOX at 20 °C	CMA vs. AOX at 25 °C
acute toxicology	0.5	>0.999	>0.999	0.922	>0.999	>0.999	0.356	0.280	0.658
	1.0	0.398	>0.999	0.012	>0.999	>0.999	0.904	0.086	0.271
	2.5	0.043	0.206	0.000	0.179	0.334	0.333	0.001	<0.0001
	5.0	0.001	0.021	0.001	0.006	0.015	0.085	<0.0001	<0.0001
	10.0	0.004	0.002	<0.0001	0.006	0.002	0.669	<0.0001	<0.0001
	20.0	>0.999	>0.999	0.016	0.724	0.515	0.329	<0.0001	0.001
long term toxicology	0.5	>0.999	>0.999	0.438	>0.999	>0.999	>0.999	0.134	0.003
	1.0	>0.999	>0.999	0.858	>0.999	>0.999	>0.999	0.064	0.064
	2.5	0.293	0.233	0.092	0.912	>0.999	0.003	0.436	0.041
	5.0	0.033	0.008	0.023	0.038	0.027	<0.0001	<0.0001	<0.0001
	10.0	0.019	0.018	0.043	0.002	0.003	0.001	<0.0001	<0.0001
	20.0	0.001	0.000	0.629	0.135	0.215	<0.0001	0.632	0.001

## Data Availability

All relevant data are within the manuscript.

## Supplementary Materials

The following are available online at https://www.mdpi.com/article/10.3390/toxics9110286/s1. Figure S1: Phase contrast pictures of CMAfin1 and AOXlar7y cell cultures with 4% ethanol supplementation directly after addition (a,c,e) and after long-term exposure (b,d,f,g) with condition of 20 and 25 °C, respectively. (h) Representative picture of the overlay mask for measuring the cell confluence of the phase contrast pictures of AOX25-4% End (g). Pictures were generated by Incucyte^®^ real-time video imaging system (Sartorius AG). Figure S2: Phase contrast pictures of CMAfin1 (a–d) and AOXlar7y (e–h) cell cultures with two isopropanol supplementation (2% and 0.125%) with 20 °C (a,b,e,f) and 25 °C (c,d,g,h) condition, respectively. Pictures were generated by Incucyte^®^ real-time video imaging system (Sartorius AG). Figure S3: Phase contrast pictures of CMAfin1 (a–h) and AOXlar7y (i–p) cell cultures with decreasing DMSO supplementation (20%, 10%, 2.5%, 1%) with 20 °C (a–d,i–l) and 25 °C (e–h,m–p) condition, respectively. Pictures were generated by Incucyte^®^ real-time video imaging system (Sartorius AG).
