# Supplementary material for: In Vitro Fish Models for the Analysis of Ecotoxins and Temperature Increase in the Context of Global Warming
Source: Toxics. 2021 Nov 1;9(11):286. doi: 10.3390/toxics9110286 (PMC8618082; doi:10.3390/toxics9110286)
Supplement: Supplementary file 1 [file toxics-09-00286-s001.zip › toxics-1380299-supplementary.pdf]

toxics

Supplementary Materials: In Vitro Fish Models for the Analysis of Exotoxins and Temperature Increase in the Context of Global Warming

Bianka Grunow, George Philipp Franz and Katrin Tönißen

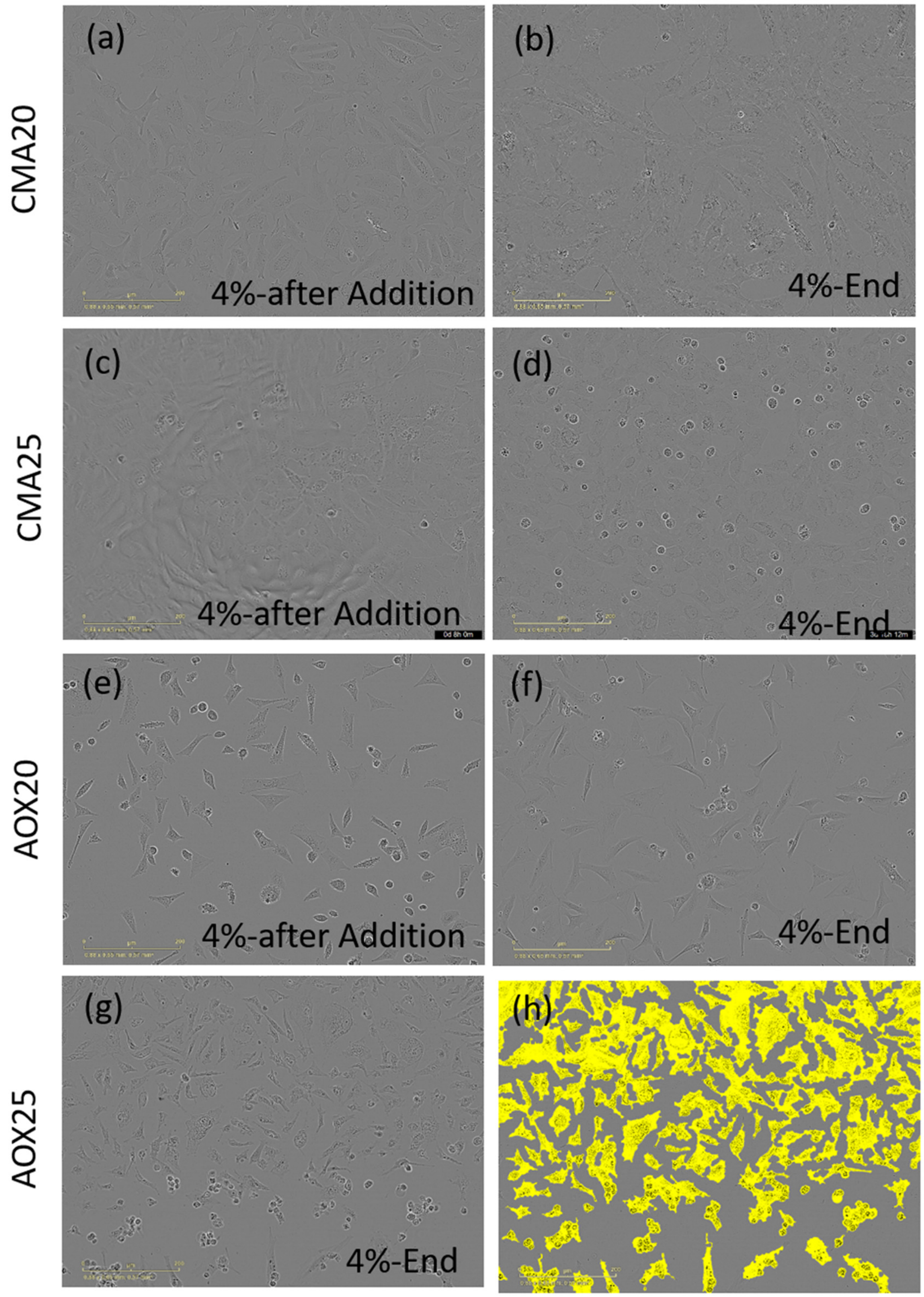

Figure S1. Phase contrast pictures of CMAfin1 and AOXlar7y cell cultures with 4% Ethanol supplementation directly after addition (a, c, e) and after long-term exposure (b, d, f, g) with condition 20 and 25 °C, respectively. (h) Representative picture of the overlay mask for meas-uring the cell confluence of the phase contrast pictures of AOX25-4% End (g). Pictures are gener-ated by Incucyte® real-time video imaging system (Sartorius AG).

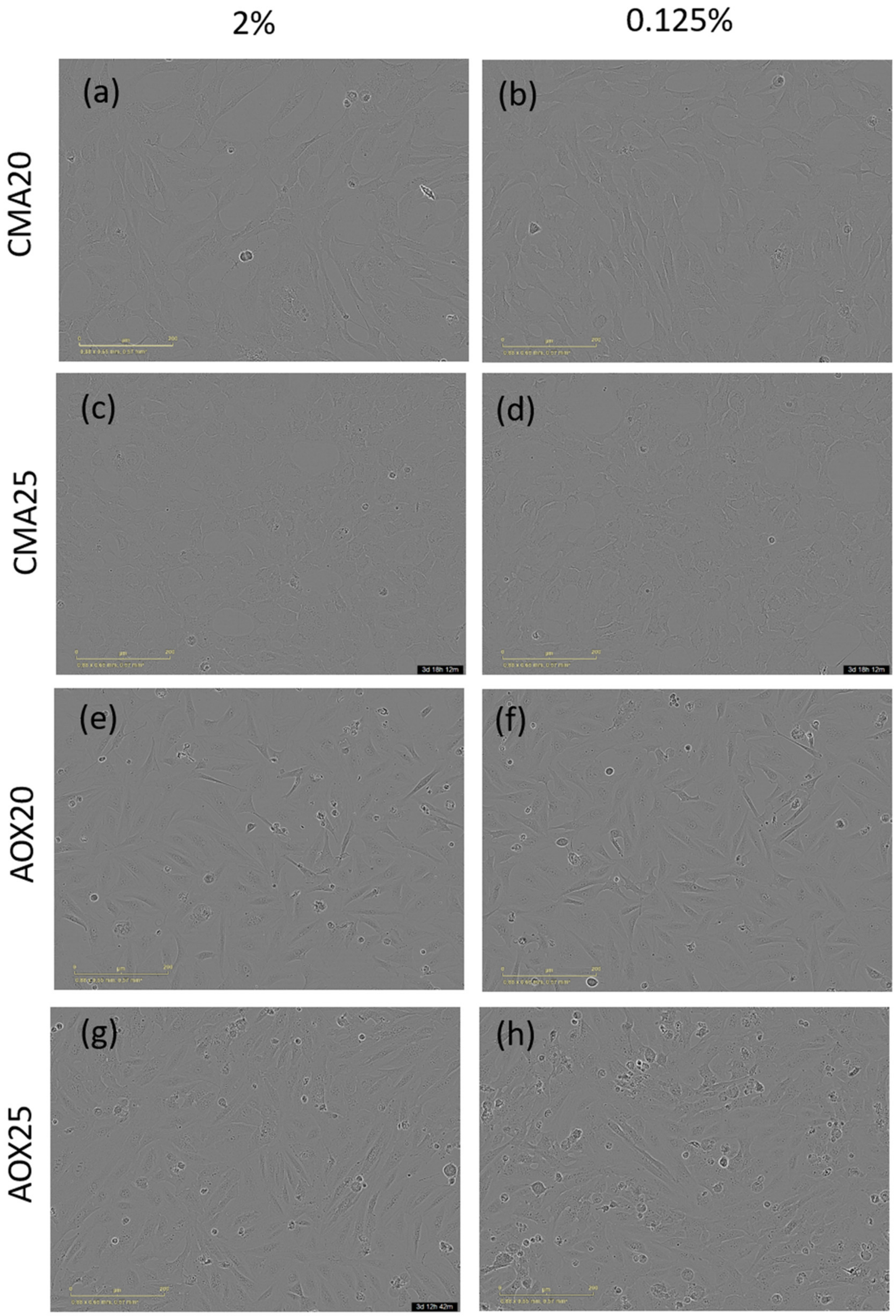

Figure S2. Phase contrast pictures of CMAfin1 (a–d) and AOXlar7y (e–h) cell cultures with two isopropanol supplementation (2% and 0.125%) with 20 °C (a, b, e, f) and 25 °C (c, d, g, h) condition, respectively. Pictures are generated by Incucyte® real-time video imaging system (Sartorius AG).

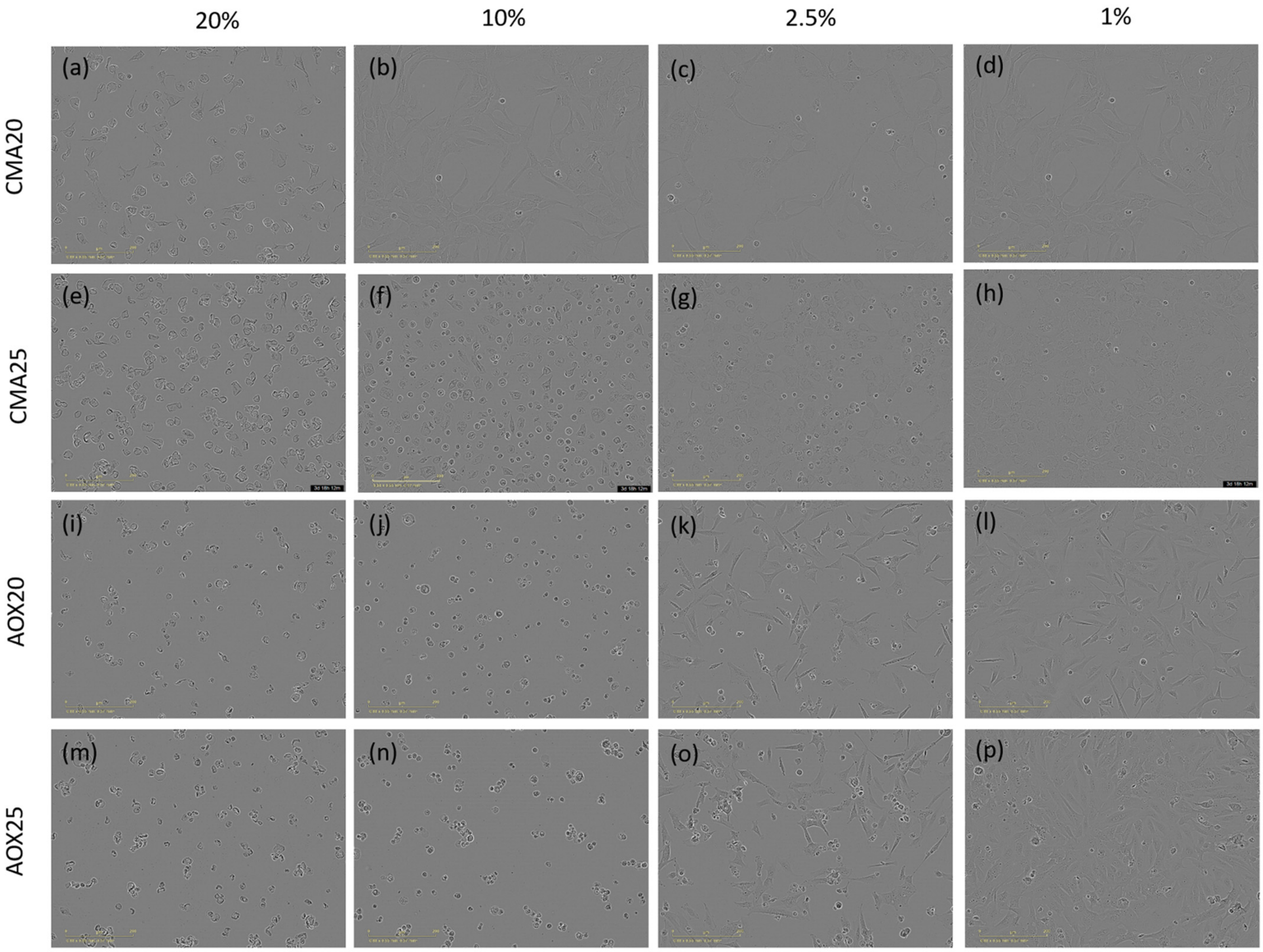

Figure S3. Phase contrast pictures of CMAfin1 (a–h) and AOXlar7y (i–p) cell cultures with decreasing DMSO supplementation (20%, 10%, 2.5%, 1%) with 20 °C (a–d, i–l) and 25 °C (e–h, m–p) condition, respectively. Pictures are generated by Incucyte® real-time video imaging system (Sartorius AG).
